# Supplementary material for: Suppression of Ribose-5-Phosphate Isomerase a Induces ROS to Activate Autophagy, Apoptosis, and Cellular Senescence in Lung Cancer
Source: Int J Mol Sci. 2022 Jul 17;23(14):7883. doi: 10.3390/ijms23147883 (PMC9322731; doi:10.3390/ijms23147883)
Supplement: Supplementary file 1 [file ijms-23-07883-s001.zip › Supplemental figures.pdf]

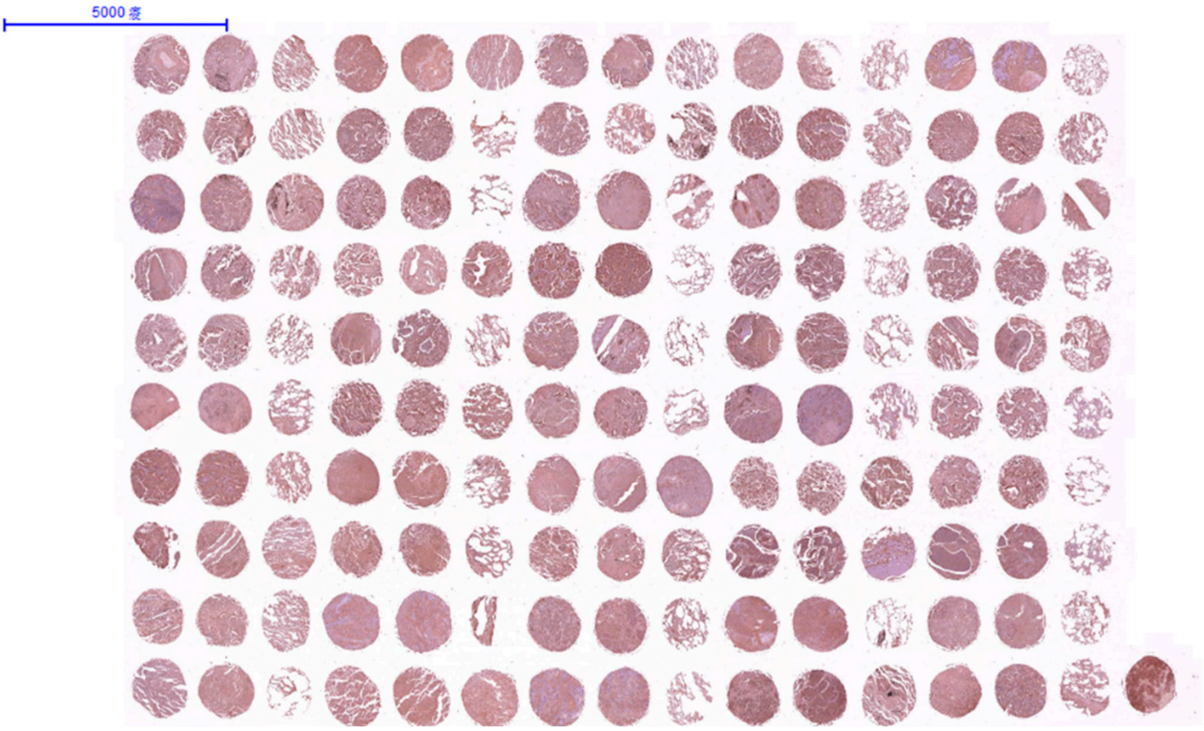

Figure S1. Complete immunohistochemistry data for RPIA in lung cancer patients' tissue array.

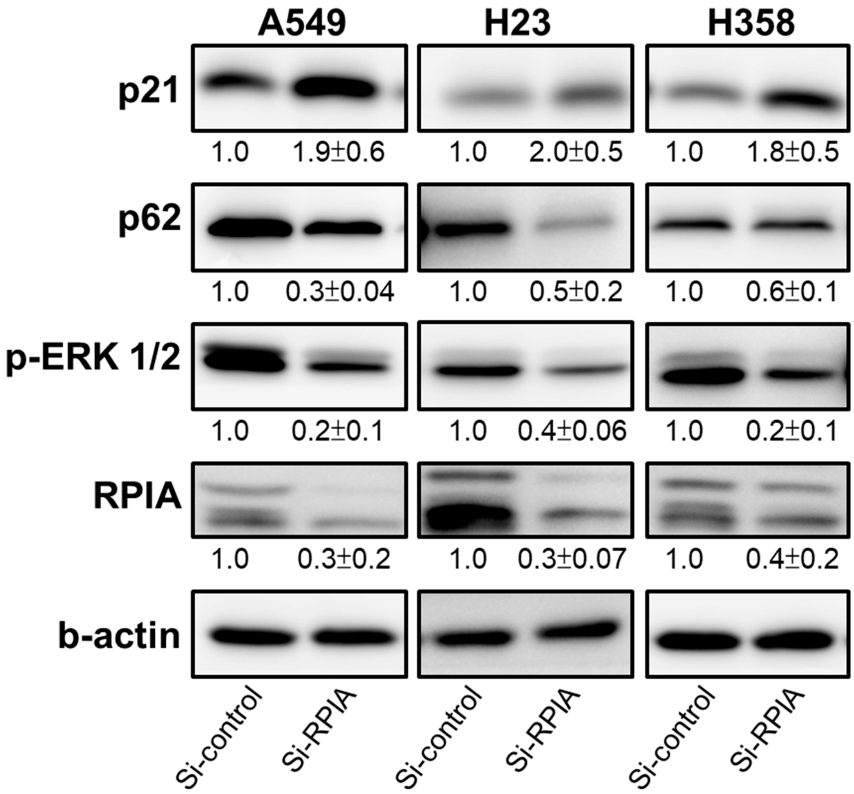

Figure S2. Knockdown of *RPIA* increases p21 and decreases p62 protein levels in lung cancer cell lines. The increased protein levels of p21 and the decreased protein levels of p62 and p-ERK1/2 were detected in A549, H23 and H358 lung cancer cells transfected with si-RPIA compared to si-control.

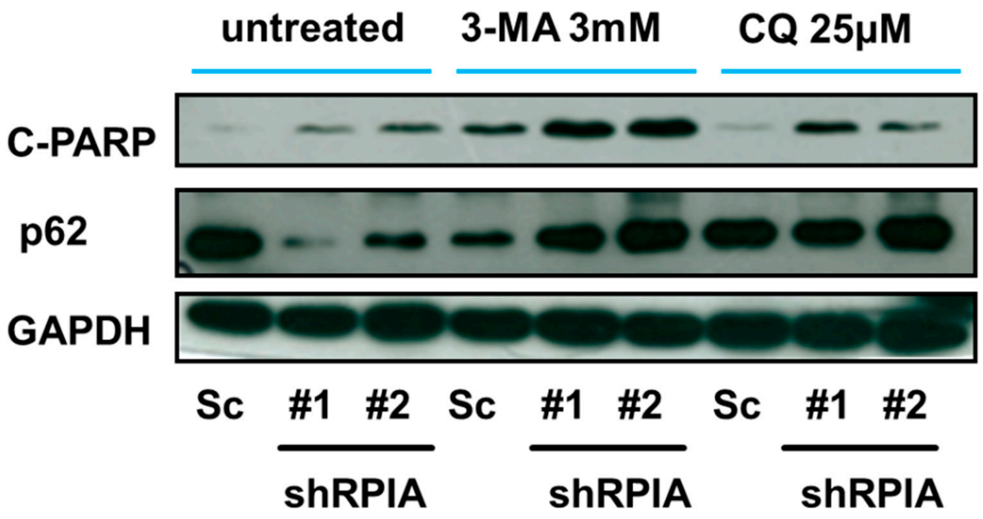

Figure S3. Inhibition of autophagy by autophagy inhibitors 3-MA and CQ further enhances the increased levels of C-PARP and reverts the decreased levels of p62 in RPIA knockdown lung cancer cells compared to the untreated group.
